# Tolllike receptor 4 (TLR4) polymorphisms in Tunisian patients with Crohn's disease: genotype-phenotype correlation

**DOI:** 10.1186/1471-230X-9-62

**Published:** 2009-08-07

**Authors:** Lilia Zouiten-Mekki, Maher Kharrat, Sami Karoui, Mariem Serghimi, Monia Fekih, Samira Matri, Lamia Kallel, Jalel Boubaker, Azza Filali, Habiba Chaabouni

**Affiliations:** 1 Department of Gastroenterology A, La Rabta Hospital – 1007, Tunisia; 2 Laboratory of Human Genetics, Medical school of Tunis, Tunisia

## Abstract

**Background:**

The immune responses to bacterial products through the pattern recognition receptor (PRR) play a pivotal role in pathogenesis of Crohn's disease. A recent study described an association between CD and some gene coding for bacterial receptor like NOD2/CARD15 gene and TLR4. In this study, we sought to determine whether TLR4 gene was associated with Crohn's disease (CD) among the Tunisian population and its correlation with clinical manifestation of the disease.

**Methods:**

90 patients with CD and 80 healthy individuals are genotyped for the *Asp299Gly *and *Thr399Ile *polymorphisms by restriction fragment length polymorphism analysis.

**Results:**

The allele and genotype frequency of the TLR4 polymorphisms did not differ between patients and controls. The genotype-phenotype correlation permitted to show that the *Thr399Ile *polymorphism was associated with early onset disease.

**Conclusion:**

this study reported the absence of association between CD and TLR4 gene in the Tunisian population, but this gene could play a role in clinical expression of the disease.

## Background

Inflammatory bowel disease (IBD) is a multifactorial polygenic disease. Some genetic markers increase the risk of ulcerative colitis (UC) or Crohn's disease (CD) while others are associated with a particular phenotypic expression like disease location and/or behaviour [1,2].

In addition to genetic predisposition, various environmental factors play a key role in the pathogenesis of IBD such as enteric bacterial flora. In IBD, a disturbed host-bacteria interaction with an aberrant mucosal immune response has been observed [3,4]. This basic immunological mechanism in IBD is demonstrated by the recently described association within IBD and gene coding for receptor of bacterial products. In 2001, several studies associated CD with NOD2/CARD15 gene which coding for cytosolic receptor of bacterial muramyl dipeptides [5-7]. The three common variants of NOD2 gene (SNP8: *Arg702Trp*; SNP12: *Gly908Arg*; SNP13: *Leu1007insC*) induce an impairment of bacterial recognition. They are associated with dysregulation of NFκB pathway and reduced α defensin release from paneth cells [8-10]. However, there are significant geographical differences in the frequency of these alleles [11]. These are not found among Japanese, Chinese, Australian and Tunisian population [12-15]; they are also less frequent in North European countries [16,17].

Toll-like receptors 4 (TLR4) is a transmembrane protein which plays a key role in bacterial lipopolysaccharides (LPS) recognition and in initiating innate immune responses [18]. It is a receptor of bacterial LPS [19]. TLR4 deficient mice are defective in their response to LPS. TLR4 is up regulated in the intestinal epithelial cells in patients with ulcerative colitis and Crohn's disease (CD) [20]. Recently, two missense polymorphisms *Asp299Gly *and *Thr399Ile *affecting the extracellular domains of TLR4 were associated with LPS hyporesponsiveness [21-23] and an association was reported between these polymorphisms and IBD [24-26].

The aim of this study was to investigate about the presence of *Asp299Gly *and *Thr399Ile *polymorphisms of the TLR4 gene among a Tunisian population with CD. We also aimed to investigate about any correlation between these polymorphisms and clinical phenotypes of CD.

## Methods

### • Subjects

The study included 90 patients with Crohn's disease, 30 patients with ulcerative colitis and 80 healthy control groups. All of them were Caucasian and the control group was matched with the patient group for sex and age.

The diagnosis of CD or UC was determined by standard clinical, radiological, endoscopic and histological criteria [27].

CD was classified according to the Vienna Classification [28]. CD patients were composed of 47 males and 43 females. Mean age was 35 years. CD was ileocolitis in 40 patients, ileitis in 22 patients and colitis in 28 patients. Mean of follow up is 90 month (24–117 month). We analysed disease behaviour at latest follow-up.

Informed consent was obtained verbally from each participant and an ethical approval was obtained by ethical committee of La Rabta Hospital.

### • Classification of the CD clinical phenotypes

We also applied an arbitrary classification based on the disease severity, need for surgery and need for immunosuppressive therapy.

The severity of CD is evaluated by the behaviour of the disease (penetrating, stricturing or inflammatory) and the installation of an acute severe colitis requiring intensive medical therapy. Within the category of severe CD, patients requiring immunosuppressive therapy or surgery were categorized under the phenotype "need for surgery" or "need for immunosuppressive".

Other phenotypic details such as disease location, age at diagnosis and presence of extra intestinal manifestations, were also recorded.

### • PCR analysis of TLR4 alleles

Genomic DNA was extracted from peripheral blood leukocytes. The two mutations of the TLR4 gene (*Asp299Gly *and *Thr399Ile*) were performed using polymerase chain reaction (PCR) and restriction fragment length polymorphism analysis (RFLP). To amplify the coding regions, we used the following primers:

for TLR4 *Asp299Gly *F: (5'AGCATACTTAGACTACTACCTCCATG3'), R: (5'GAGAGATTTGAGTTTCAATGTGGG3')

and for TLR4 *Thr399Ile *F: (5'GGTTGCTGTTCTCAAAGTGATTTTGGGAGAA3'), R: (5'GGAAATCCAGATGTTCTAGTTGTTCTAAGCC3').

The PCR was carried out by 30 cycles of denaturing at 94°C for 1 min, annealing at 55°C (*Asp299Gly*) and at 53°C (*Thr399Ile*) for 1 min, and extension at 72°C for 1 min. PCR products were cleaved overnight with *Nco I *for *Asp299Gly *polymorphism and *HinfI *for *Thr399Ile *polymorphism (Biolabs) at 37°C and electrophored on a 2,5% agarose gel. The fragment sizes for carriers of the polymorphic allele decreased from 188 bp (wild type: alleleA) to 168 bp (allele G) for the *Asp299Gly *polymorphism and from 124 bp (wild type: allele C) to 98 bp (allele T) for the *Thr399Ile *polymorphism.

### • Statistical analysis

Chi square test was used to compare the allele and genotype frequencies among disease and control groups. To correlate phenotype genotype state, the T-student test was used for quantitative parameters and Fischer test for qualitative parameters. The Kaplan-Meir with Log-Rank test was used to compare the delay of surgery within the different groups. P values less than 0.05 were considered significant.

## Results

Genotype frequency and allele distribution of TLR4 *Asp299Gly *polymorphism are summarized in table 1. The genotype GG was not found in both patients and control groups. The frequencies of the 299Gly allele were respectively, 7,5% and 6% in CD and healthy controls. No significant difference was noticed in the *Asp299Gly *polymorphism frequencies among CD patients and controls.

**Table 1:** TLR4 *Asp299Gly *polymorphism: Genotype and allele frequencies (%) of in Crohn's disease (CD) patients and controls.

	CD (n = 90)	Controls (n = 80)	P
Genotype			
AA	86% (78)	89% (71)	0.6
AG	14% (12)	11% (9)	
GG	0	0	

Alleles			
A	93% (168)	94% (151)	0.6
G	7% (12)	6% (9)	

TLR4 *Thr399Ile *genotype carrier frequencies are summarized in table 2. None patients and control, were homozygote for allele T. The allele T was more frequent in CD patient than controls (7% and 4,5% respectively) but the difference was not significant. The distribution of *Thr399Ile *polymorphism was similar in UC group, CD and controls.

**Table 2:** TLR4 *Thr399Ile *polymorphism: Genotype and allele frequencies (%)in Crohn's disease (CD) patients and controls.

	CD (n = 90)	UC (n = 30)	Controls (n = 80)	P
Genotype				0.38
CC	86% (77)	93% (28)	90% (72)	
CT	14% (13)	7% (2)	10% (8)	
TT	0	0	0	
Alleles				
C	93% (167)	97% (58)	95% (152)	0.5
T	7% (13)	3% (2)	5% (8)	

The correlation study between *Asp299Gly *polymorphism and phenotype of CD didn't either allow to associate TLR4 genotype with specific disease behaviour, or with the severity of CD. Whereas, for *Thr399Ile *polymorphism, we noted an earlier onset of disease in CD patients carrying allele T (27 years ± 9 vs 36 years ± 10). This difference is statistically significant (p = 0.01) (table 3).

**Table 3:** TLR4 alleles and clinical characteristics of CD patients.

	Asp299Gly		Thr399Ile	
	G(%)	A(%)	T(%)	C(%)
Sex				
M	9	43	9,5	41,5
F	7	41,5	5	44
Age (years)	30 ± 12	36 ± 12	27 ± 9	36 ± 10*
Location				
Ileum	5	19	1	21
Ileocolic	6	37	6	37
Colon	3	28	7	27
Anoperineal manifestations	2	28	2	28
Penetrating and/or Stricturing form	11	47	9,5	50
Acute severe colitis	3	12	3,5	15
Need for immunosuppressive	8	31,4	7	36

*: p = 0.01

**Figure 1:**
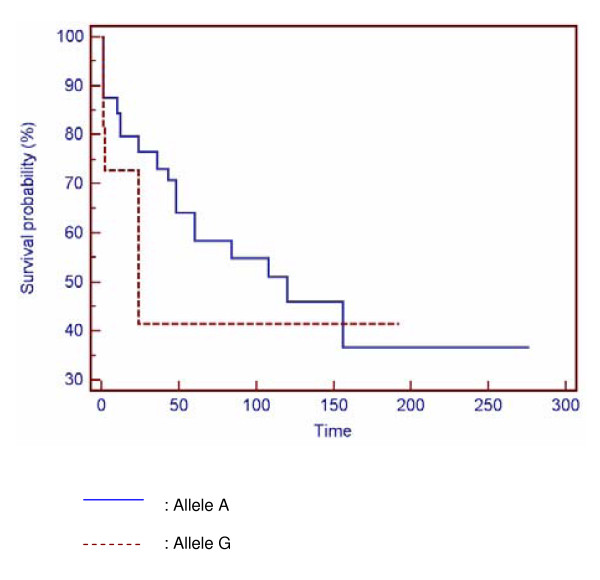
**Comparison of the delay of surgery between allelic variants of *Asp299Gly *polymorphism**.

**Figure 2:**
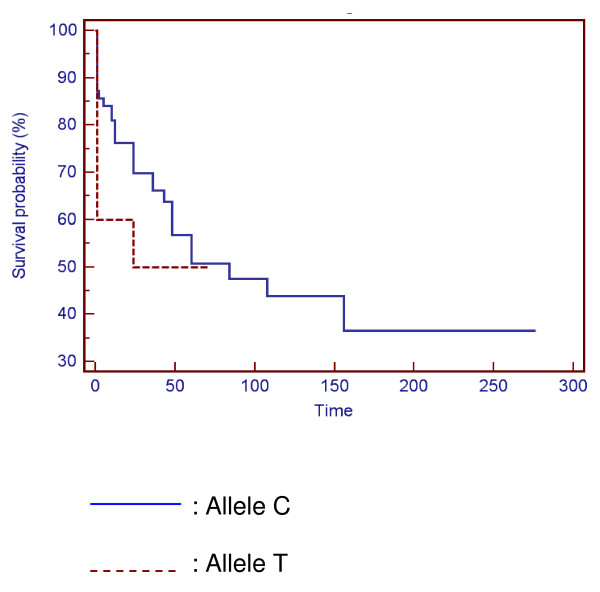
**Comparison of the delay of surgery between allelic variants of *Thr399Ile *polymorphism**.

Different analyses, based on the time between the diagnosis of the complication and the onset of the disease: stricturing or penetrating form, acute severe colitis, need for surgery or immunosuppressive therapy, have been studied. No significant differences were noticed between these parameters and genotype (Figure 1 and Figure 2).

The extraintestinal manifestations are observed in 20% of patients but only three of them have TLR4 mutations.

We also evaluated the co-existence of TLR4 mutations and those of NOD2 and HSP70-2 genes in CD patients (previous studies for the same patients) [12-29]. The number of patients carrying a TLR4 mutations and one of NOD2 and/or HSP70-2 mutations, was 20 (19%) vs 17% in controls subjects. Statistically, no difference was noticed between patients and controls.

The co-existence of TLR4 mutations was associated with prevalent complications of CD (73% vs 27% p = 0.06). Three of CD patients were carrying simultaneously a polymorphic allele of the three tested genes. These patients have a severe form of the disease.

Additionally, 76% of the CD patients were carrying one mutation of TLR4 gene, in minimum, compared to 65% in controls group (p = 0.09).

## Discussion

Following the identification of the NOD2/CARD15 gene in CD, the role of innate immune system in the pathogenesis of IBD has gained an increasing attention. Compared to mutations in the cytosolic receptor NOD2/CARD15, the TLR4 polymorphisms caused an impaired response to LPS [30]. Cario and Podolsky showed that TLR4 was strongly up-regulated in CD and UC [20], this may be caused by an exaggerated host defence reaction of the intestinal epithelium to endogenous luminal bacterial flora [31].

The importance of TLR4 polymorphisms in CD is less clear than NOD2/CARD15. In healthy Caucasians, the reported frequencies of these polymorphisms ranges are between 7% and 12% [32].

Franchimont et al. have reported a two-fold elevation in allele frequency of TLR4 *Asp299Gly *in CD Belgium patients [26]. The Thr399Ile variant was associated with UC in German study [24]. Japanese [30] and Hungarian studies [33] failed to detect any individuals displaying the mutant alleles in TLR4. There is also no difference in TLR4 allele frequency between IBD patients and controls in preliminary results of the EC-IBD study group [17].

These finding comfort our results, since in our study, about TLR4 polymorphisms, no significant differences in the allele or genotype frequencies between the study groups were noticed. The small size of our cohort may contribute to these negative results.

The correlative study between TLR4 mutations and CD, allowed us to show an early disease onset in the subgroup of patients, carrying a *Thr399Ile *variant in our study (27 years ± 9 vs 36 years ± 10; p = 0.01).

In literature, the presence of variant TLR4 allele was tendency to be associated with an early disease onset in Hungarian study (p = 0.06); the earliest onset was observed in carriers of both variant NOD2/CARD15 and TLR4 alleles (23 years vs 30.2 years; p = 0.01)[33]. These results differ from those of another study that doesn't establish any association between TLR4 polymorphisms and the age of the disease onset [34]. This discrepancy of results necessities more study with a large cohort.

Ouburg et al. [35] described an association between the TLR4 polymorphisms and colonic localisation (43% vs 12%; p = 0.0017). This association could not be confirmed in our study.

The three candidate genes of CD: NOD2, HSP70-2 and TLR4, studied in the Tunisian population, were not associated with the disease among our population, contrarily to European and American results [29,36-38].

The co-existence of TLR4, NOD2/CARD15, HSP70-2 mutated alleles is distributed similarly in CD and control group. It seems that these genes don't increase the risk of developing the disease, but they play probably a role in phenotype determines, since more complications were noticed within patients carrying two or more mutations (p = 0.06).

Three patients were found to carry mutations in all three genes. These patients had an earlier disease onset (19, 23 et 30 years), an ileocolitis location, a stricturing perforating form and anoperinal manifestations and then, a severe form of the disease.

Analysing the SNP interaction will allow an understanding of the phenotypic variation of the disease, but until now the results in CD are controversial. In fact, some studies show that the coexistence of mutations of NOD2/CARD15 and TLR4 genes, increase the risk of developing CD [39-43] or, is associated with the phenotype of the disease, but these results were not confirmed.

## Conclusion

In conclusion, our study is unable to detect an association within the TLR4 gene and CD, but this gene could be implicated in the phenotype of the disease, mainly with early onset of disease. The co-existence of several allelic variant of TLR4, NOD2/CARD15 and HSP70-2 genes may be associated with severe form of the Crohn's disease among the Tunisian population.

The association between NOD2/CARD15, HSP70-2 and TLR4 mutations with CD varies from a population to another; within the USA, European, Asiatic or the Tunisian population. Further genetic studies should definitely resolve the impact of these genes in susceptibility to CD, depending on the ethnic background.

## Abbreviations

CD: Crohn's disease; PCR: polymerase chain reaction.

## Competing interests

The authors declare that they have no competing interests.

## Authors' contributions

LMZ carried out the molecular genetic studies, participated in the recruitment of the patients and drafted the manuscript. M K participated in the molecular genetic studies. SK performed the statistical analysis and participated to the recruitment of patients. MS, MF, LK, SM and JB participated in the recruitment of patients. AF and HC conceived of the study, participated in its design and coordination and helped to draft the manuscript. All authors read and approved the final manuscript.

## Pre-publication history

The pre-publication history for this paper can be accessed here:

http://www.biomedcentral.com/1471-230X/9/62/prepub
